# Multiyear wildfire smoke exposure (MultiWiSE) metrics: a data-driven approach to characterizing episodic PM_2.5_ exposures for epidemiologic research

**DOI:** 10.1038/s41370-026-00876-5

**Published:** 2026-04-13

**Authors:** Stephanie E. Cleland, Olivia Pearl Xi-Qiong Hamilton, Michael Brauer, Sarah B. Henderson

**Affiliations:** 1 Faculty of Health Sciences, Simon Fraser University, Burnaby, BC Canada; 2 Legacy for Airway Health, Vancouver Coastal Health Research Institute, Vancouver, BC Canada; 3 Environmental Health Services, BC Centre for Disease Control, Vancouver, BC Canada; 4 School of Population and Public Health, University of British Columbia, Vancouver, BC Canada

**Keywords:** Wildfires, Exposure assessment, Particulate Matter, Air Pollution, Analytical Methods

## Abstract

**Background:**

Population exposure to wildfire smoke (WFS) has increased across North America. The health effects of short-term WFS exposure are widely documented, but little is known about longer-term exposures. Most epidemiologic studies use multiyear averages to characterize long-term air pollution exposure, but these do not reflect the episodic nature of WFS which may be associated with distinct health risks.

**Objective:**

We developed twelve data-driven Multiyear Wildfire Smoke Exposure (MultiWiSE) metrics that characterize the frequency, intensity, and duration of episodic WFS for application in epidemiologic studies examining the effects of longer-term exposures.

**Methods:**

The MultiWiSE metrics are easily calculable using any long time series of daily fine particulate matter (PM_2.5_). The approach first establishes a location-specific counterfactual, which is used to separate WFS from non-WFS PM_2.5_. Estimates of weekly WFS PM_2.5_ are then used to generate the metrics. We demonstrate this approach in British Columbia (BC), using PM_2.5_ estimates from the Canadian Optimized Statistical Smoke Exposure Model for 2010-2023.

**Results:**

Of the MultiWiSE metrics, two describe cumulative exposure, four describe WFS-impacted weeks, five describe WFS episodes, and one describes recovery. When applied to 652 BC census subdivisions, WFS accounted for 1.7–24.5% of cumulative PM_2.5_ exposure, with a mean (range) of 45.2 (17–80) WFS-impacted weeks and 8.2 (3–15) WFS episodes. WFS episodes lasted up to 24 weeks, with an average recovery of 78.2 (41.1–178.5) weeks between episodes. Eleven metrics were positively correlated, with correlations ranging from 0.20 to 0.99 and a mean of 0.70, indicating they capture both overlapping and distinct features of multiyear WFS exposure.

**Significance:**

The MultiWiSE metrics characterize the frequencies, intensities, and durations of episodic WFS exposure and can be calculated using any multiyear time series of PM_2.5_. They can be used in epidemiologic studies for a more nuanced and actionable understanding of the health risks of longer-term exposures.

**Impact statement:**

- Population exposure to wildfire smoke (WFS) has increased across North America. While the health effects of short-term exposure are widely documented, little is known about the effects of longer-term exposure. Most studies use multiyear averages to characterize long-term air pollution exposure, which do not capture the episodic nature of WFS. To support epidemiologic research on multiyear exposures, we developed twelve Multiyear Wildfire Smoke Exposure (MultiWiSE) metrics that can be calculated using any long time series of daily fine particulate matter. These data-driven metrics characterize the frequency, intensity, and duration of episodic WFS exposure across large and variably smoke-impacted regions.

## Introduction

Wildfire activity is becoming more frequent and intense in many areas of the world, partially due to climate change [1]. As a result, populations near to and distant from wildfires are being exposed to more wildfire smoke (WFS), with longer and more extreme exposures within wildfire seasons, and more frequent exposures across wildfire seasons [2–5]. Fine particulate matter (PM_2.5_), a primary component of WFS, is commonly used to characterize WFS exposure. PM_2.5_ has been associated with a wide range of health effects in response to both short- and long-term exposures [6], is clearly elevated during WFS-impacted periods [7], and is widely measured [8].

There is a rapidly growing body of literature on short-term exposure to WFS PM_2.5_ and health effects ranging from birth outcomes to premature mortality [9]. Although studies on multiple endpoints are still limited, there is strong evidence that the adverse effects of short-term exposure to WFS PM_2.5_ are consistent with the effects of short-term exposure to PM_2.5_ from other sources [9–12]. Additionally, some epidemiologic studies have indicated that the effects of WFS PM_2.5_ are larger than those of PM_2.5_ from other sources [13, 14]. These larger effects are supported by toxicological evidence [15] and may be due to differences in PM_2.5_ composition, size distribution, atmospheric aging, unmeasured co-pollutants, or a combination of these factors [16]. The observed differences underscore the importance of using estimates of WFS PM_2.5_ to understand the health effects specific to WFS.

There is also growing evidence on the health effects of longer-term WFS exposure, though the body of relevant literature is much smaller [17, 18]. These studies have primarily characterized exposure using annual or multiyear averages of WFS PM_2.5_, and the reported effects include decreased academic performance [19], decrements in lung function [20], increased incidence of respiratory infections [21], heart disease [22], dementia [23], cancer [24], and non-accidental mortality [25]. Once again, this literature on WFS PM_2.5_ is generally consistent with the much larger literature on PM_2.5_ from other sources. However, patterns of WFS exposure are distinct from other sources of air pollution, and more studies are needed to better elucidate the health risks of prolonged and repeated exposure.

One critical challenge in studying the health effects associated with multiyear exposure to WFS PM_2.5_ lies in characterizing those exposures, which differ from exposure to other sources of PM_2.5_ in three important ways. First, WFS is highly episodic in both space and time due to variation in the location and timing of emissions and the impact of meteorology. Any given location may have significant exposures in some wildfire seasons and none in others. While urban sources of PM_2.5_ can also be episodic and temporally variable, these sources tend to be more constant and spatially uniform, with episodes driven by meteorological phenomena. Second, the magnitude of WFS PM_2.5_ can be highly variable within wildfire seasons and from year to year. While extreme events receive a lot of attention, the lower-magnitude exposures that happen more frequently may be associated with higher health burdens [26]. Finally, the episodic nature of WFS exposure leads to long periods of non-exposure between active wildfire seasons. For some health effects, these periods may allow for biological recovery from damages caused by WFS, which may differ from the damages caused by air pollution from more constant sources, such as traffic or industry.

The episodic and sometimes extreme nature of WFS PM_2.5_ may also lead to exposures with potentially different biological impacts than those from non-WFS PM_2.5_. For example, severe WFS episodes coinciding with specific periods of pregnancy or infant development may lead to developmental changes with long-lasting sequelae [27]. This was observed in a cohort of rhesus macaque monkeys, where those exposed to WFS during infancy showed reduced lung function by age 3 [28]. Together, these differences between WFS and other sources of air pollution suggest a need to look beyond the annual or multiyear PM_2.5_ averages when characterizing WFS for studies on the health effects of longer-term exposure.

The spatiotemporal variability in WFS can also lead to different exposure patterns at different locations [18]. Consider the example of three places with the same cumulative exposure to WFS PM_2.5_ over a multiyear period. One receives the exposure from extreme WFS episodes during a few wildfire seasons, one receives more moderate exposures in multiple seasons, and one receives smaller exposures almost every season. While these locations are exposed the same total amount of WFS during the multiyear period, the nature of how they were exposed is distinctly different. Although it is likely that these different WFS exposure profiles will lead to different biologic and behavioral responses, at this stage of the literature, we do not know how patterns of WFS exposure influence the associated health risks. To address this knowledge gap, we need ways to better characterize the health effects of episodic WFS exposures over multiple years.

**Fig. 1:**
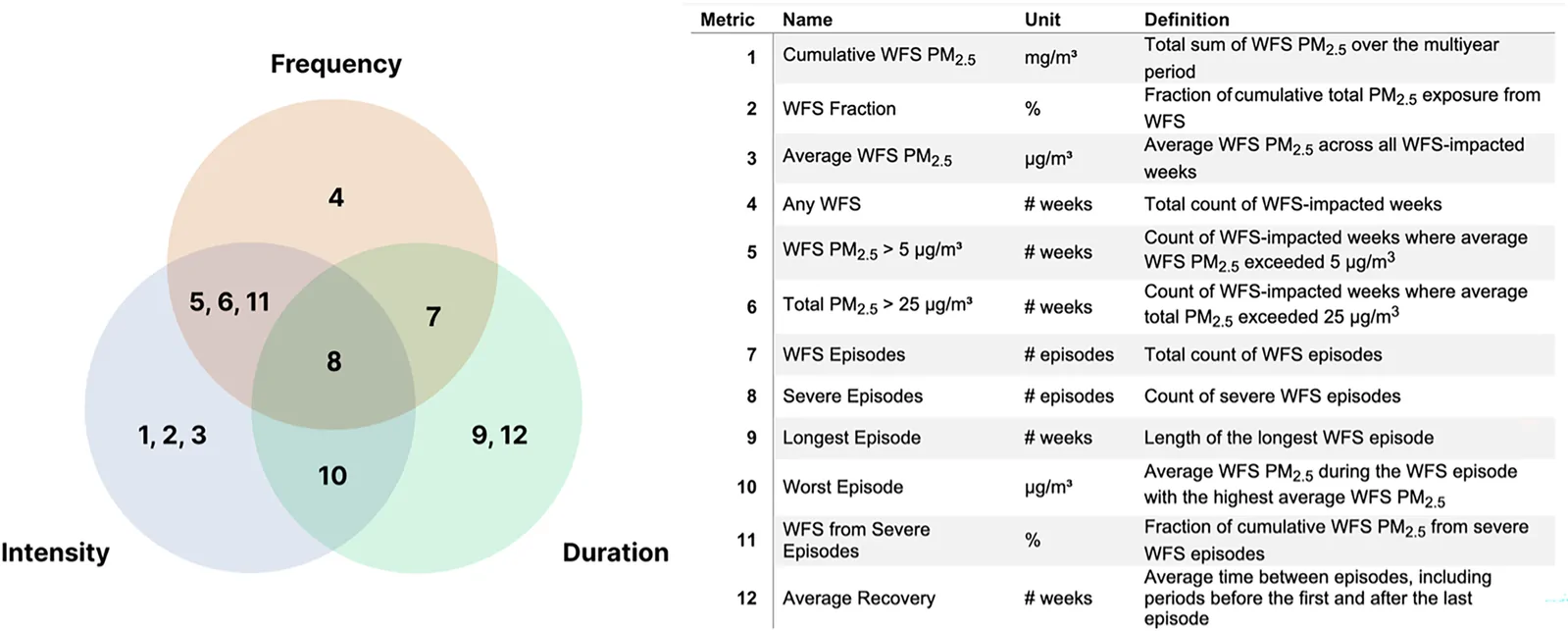
The twelve Multiyear Wildfire Smoke Exposure (MultiWiSE) metrics that capture the frequency, intensity, and duration of episodic exposure to wildfire smoke (WFS) fine particulate matter (PM_2.5_). Adapted from Casey et al. [30].

Here we introduce twelve Multiyear Wildfire Smoke Exposure (MultiWiSE) metrics, which have been developed to support a national program of research on the health effects of multiyear WFS exposure in Canada. The MultiWiSE metrics build on ideas established by other research groups [18, 29–31] and characterize the following features of WFS PM_2.5_ exposure: intensity, duration, and frequency (Fig. 1). The MultiWiSE metrics were designed to be easily calculable using any long time series of daily total (i.e., all-source) PM_2.5_ measurements or estimates, without the need to consider other data on wildfires, smoke, or atmospheric conditions. Therefore, one key objective of the proposed data-driven approach was to differentiate WFS PM_2.5_ from non-WFS PM_2.5_ using only the time series of total PM_2.5_ concentrations at a given location. Estimates of both total PM_2.5_ and WFS PM_2.5_ are used to calculate the MultiWiSE metrics, but with simple modifications, the metrics can also be easily applied to a long time series of daily WFS PM_2.5_ estimates. Thus, they can be readily applied to any multiyear period in any location by users who may not be familiar or adept with the other types of geospatial data often used in WFS exposure assessment. The metrics were also designed to be simple, with a focus on easy interpretability to facilitate public health communication about the health risks of multiyear WFS exposure. In this paper, we demonstrate application of the MultiWiSE metrics across the Canadian province of British Columbia (BC), and to the three hypothetical locations described above.

## Methods

### Study setting and period

The MultiWiSE metrics were developed and applied in BC, which is the westernmost province in Canada. The province is heavily forested with complex topography, defined by the Coast Mountains on the west coast, the Rocky Mountains along the eastern border with Alberta, and the dry interior plateau between them. Like other regions on the west coast of North America, BC has a long history of severe wildfire seasons with a marked increase in wildfire activity over the past 20 years [32]. It provides an ideal setting to test the MultiWiSE methods due to the significant spatiotemporal variation in WFS and generally low background PM_2.5_ concentrations.

Analyses were conducted at the level of the national census subdivision, of which there were 652 in the 2016 census that were populated and had PM_2.5_ data available. Census subdivisions are relatively stable geographic areas with highly variable population density depending on their urbanicity. The analyses covered the 14-year period from 2010 to 2023, or 730 consecutive weeks, with above-average wildfire seasons in 2010, 2014, and 2015, and severe wildfire seasons in 2017, 2018, 2021, and 2023 [32]. The wildfire season is defined here as May 1 through October 31, which captures the vast majority of wildfire activity in BC while largely excluding controlled burning and residential woodsmoke in the shoulder seasons [33].

### PM_2.5_ data

Daily estimates of total (i.e., all-source) PM_2.5_ were taken from the Canadian Optimized Statistical Smoke Exposure Model (CanOSSEM), which is described in detail elsewhere [34]. Briefly, CanOSSEM combines PM_2.5_ measurements with satellite observations of wildfires, aerosol, and meteorological parameters to generate daily estimates of average PM_2.5_ at a 5 km x 5 km resolution for all populated areas of Canada. The random forest model performs well, with a root mean squared error (RMSE) of 2.96 μg/m^3^ and 96% of estimates within 5 μg/m^3^ of the observed values for the original 2010-2019 training period [34]. With the inclusion of data from 2020 to 2023, the RMSE increased to 3.73 μg/m^3^, and 96% of estimates still remained within 5 μg/m^3^ of the observed values (unpublished data). The increased RMSE was largely due to the unprecedented 2023 wildfire season across Canada [32].

Data from CanOSSEM have been used for multiple epidemiologic studies [35–37] and will provide the foundation for a national program of research on chronic health effects associated with WFS PM_2.5_. For these analyses, we first aggregated the daily CanOSSEM PM_2.5_ estimates to the census dissemination area-level, of which there were 7,368 in the 2016 census that were populated and overlapped with the available PM_2.5_ estimates. Dissemination areas have a population of approximately 400-700 persons and are the smallest geographic unit at which census results are reported. Each dissemination area maps to a single census subdivision, which represents municipalities or municipal equivalents in Canada. Next, we calculated daily population-weighted PM_2.5_ estimates for the 652 census subdivisions. Weekly sums and averages of daily PM_2.5_ were then calculated for each census subdivision by epidemiologic week, where the first epidemiologic week of each year starts on the first Sunday of the year [38]. Only complete epidemiologic weeks between 2010 and 2023 were used for analysis, leading to 730 consecutive weeks between January 3, 2010, and December 30, 2023. All 652 census subdivisions had PM_2.5_ data available for 99.5% or more of the 730 weeks.

### Establishing the location-specific counterfactual and separating WFS from non-WFS PM_2.5_

The methods described here were designed to require only a multiyear time series of daily total PM_2.5_ estimates or measurements, so they can be easily applied in any location without the need for other complex datasets. Similar to O’Dell et al. [29], the approach is predicated on determining the normal range of PM_2.5_ concentrations in the absence of WFS during the multiyear period of interest. Once established, we use that baseline to define a counterfactual PM_2.5_ concentration for WFS-impacted weeks (i.e., the PM_2.5_ concentration expected in the absence of WFS), then separate total PM_2.5_ into its non-WFS PM_2.5_ and WFS PM_2.5_ components. The following section describes the process in detail (also see Fig. S1).

**Fig. 2:**
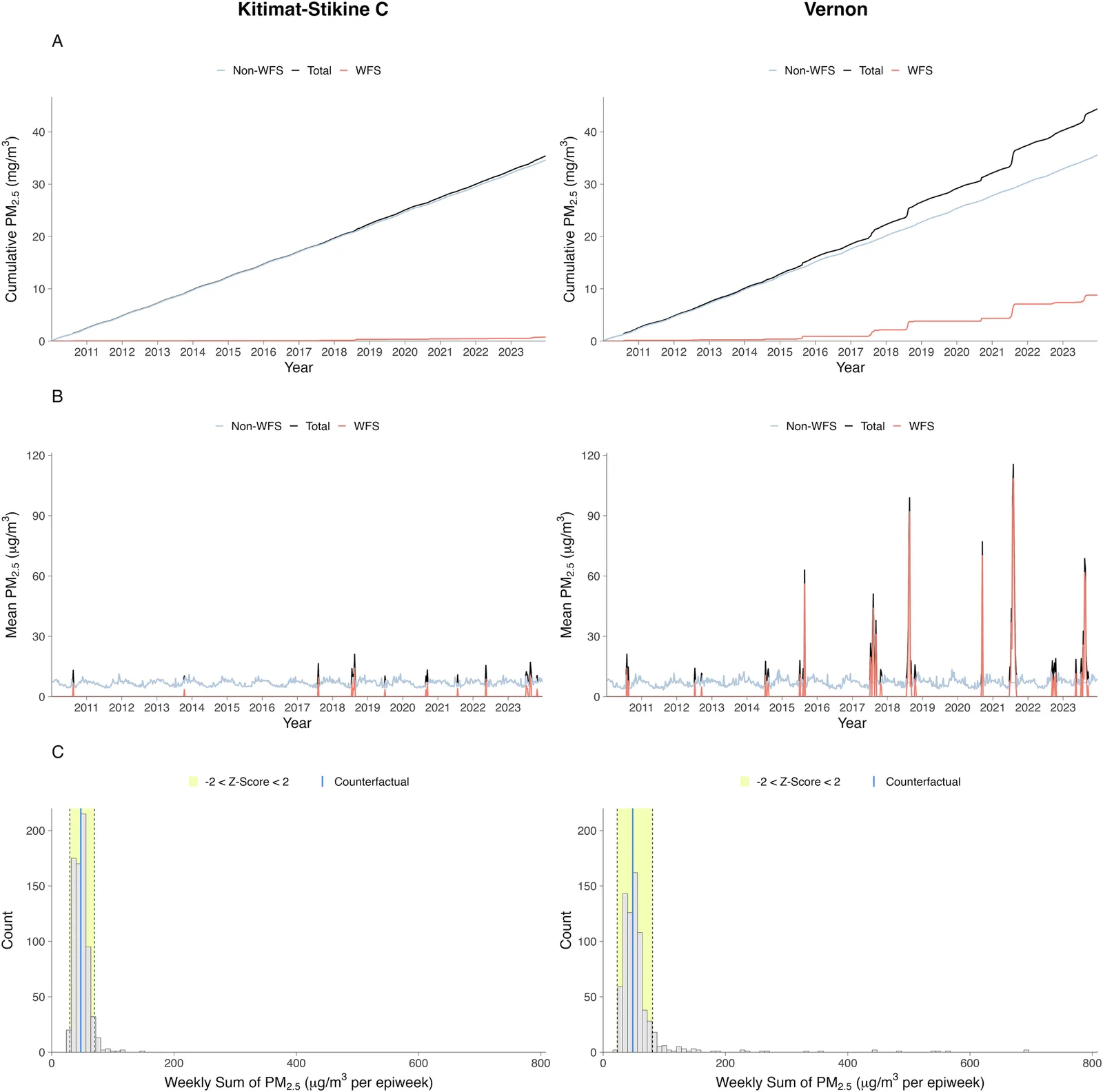
Wildfire smoke (WFS) exposure profiles for two census subdivisions in British Columbia for 2010–2023. The panels display the **A** cumulative and **B** average weekly total, WFS, and non-WFS fine particulate matter (PM_2.5_) and **C** distribution of the weekly sum of total PM_2.5_ for each census subdivision. The distribution plots highlight the range and median of normal weekly sums (modified Z-score between −2 and +2), which are used to establish the counterfactual value and extract the WFS and non-WFS contributions from total PM_2.5_. The locations of the two census subdivisions were chosen to indicate low (Kitimat-Stikine C) and high (Vernon) WFS impacts, and are shown in Fig. S2. The Multiyear Wildfire Smoke Exposure (MultiWiSE) metrics for the two locations can be found in Table S1.

First, daily total PM_2.5_ is used to calculate the sum and average of PM_2.5_ for each epidemiological week in the multiyear period. Next, the distribution of the weekly sum of total PM_2.5_ is used to identify all values with modified z-scores ranging from −2 to +2. The modified z-score is a robust alternative to the z-score when working with skewed data [39]. The median of these values is set as the counterfactual value, meaning the weekly sum of PM_2.5_ expected in the absence of WFS. Next, weeks during the wildfire season (May 1 to October 31 used here) with a modified z-score greater than +2 are identified as WFS-impacted, indicating that one or more days during that week were affected by WFS. For WFS-impacted weeks, the non-WFS PM_2.5_ is set to the counterfactual value, and the WFS PM_2.5_ is set to the difference between total PM_2.5_ and non-WFS PM_2.5_. For non-WFS-impacted weeks, the total PM_2.5_ is retained as the non-WFS PM_2.5_ and WFS PM_2.5_ is set to zero. The total PM_2.5_, non-WFS PM_2.5,_ and WFS PM_2.5_ are then used to create cumulative exposure trajectories using weekly sums and exposure time series using weekly averages, where the weekly sums are divided by 7 (Fig. 2 shows examples at two locations).

### Multiyear wildfire smoke exposure (MultiWiSE) metrics

Once the location-specific counterfactual has been established and the weekly averages and sums of WFS PM_2.5_ and non-WFS PM_2.5_ have been calculated, the estimates of WFS PM_2.5_ can be used to generate the twelve MultiWiSE metrics to comprehensively characterize exposure to WFS (Fig. 1). The MultiWiSE metrics are calculated using weekly PM_2.5_ to balance the need for a time scale suited to studying the health effects of multiyear exposure with the ability to detect short-term peaks in PM_2.5_. The metrics fall into four groups described as cumulative exposure, weekly exposure, episode exposure, and recovery, described in more detail below. Here, the metrics are calculated for 2010–2023, but this exposure window can be flexibly defined to capture any time period of interest (e.g., exposure in the 5 years prior to a health outcome). The MultiWiSE metrics can also be used to examine spatial and temporal trends in WFS exposure, which we demonstrate by comparing the metrics across BC census subdivisions for the entire study period and for the first and last 7 years. Once the twelve MultiWiSE metrics are calculated, Spearman correlation coefficients can be used to evaluate how well the metrics capture different features (frequency, intensity, and duration) of WFS PM_2.5_ exposure.

Cumulative exposure: These two metrics describe cumulative exposure to WFS over the entire exposure window, reflecting overall intensity of the exposure. **Metric 1** is cumulative WFS PM_2.5_, which reflects the total sum of exposure over the multiyear period in mg/m^3^. It is calculated by adding together the weekly sums of WFS PM_2.5_ and represents the sum of daily average WFS PM_2.5_ across the entire period. **Metric 2** is the WFS fraction of the cumulative total PM_2.5_ exposure (%), calculated by dividing the cumulative WFS PM_2.5_ by the cumulative total PM_2.5_.

Weekly exposure: These four metrics describe WFS-impacted weeks, defined as weeks during the wildfire season with a modified z-score greater than +2. **Metric 3** is the average WFS PM_2.5_ concentration (μg/m^3^) across all WFS-impacted weeks. This metric is comparable to the annual or multiyear averages typically used in epidemiologic studies of long-term air pollution exposure. **Metric 4** is any WFS, defined as the total count of WFS-impacted weeks (i.e., weeks with any WFS PM_2.5_) during the exposure window, and **Metric 5** is the count of WFS-impacted weeks when the weekly average WFS PM_2.5_ is greater than 5 μg/m^3^, chosen to be consistent with the definition used by Casey et al. [30]. **Metric 6** is the count of WFS-impacted weeks when the weekly average total PM_2.5_ is greater than 25 μg/m^3^, suggesting that on average, the 24 h PM_2.5_ concentrations were greater than 25 μg/m^3^ over the 7-day period, in part due to WFS. This value was chosen because it is the current 24 h air quality objective in BC, though any similar daily regulatory value could be substituted in its place (e.g., 35 μg/m^3^ is the National Ambient Air Quality Standard in the US).

Episode exposure: These five metrics describe WFS episodes, considering both all episodes and severe episodes. WFS episodes (i.e., all episodes) were defined to capture both short periods with extreme WFS as well as longer periods with more moderate WFS, and severe episodes were defined to isolate the more extreme WFS episodes. A WFS episode is defined as either (1) two or more WFS-impacted weeks separated by no more than three non-WFS-impacted weeks, or (2) a single WFS-impacted week where the sum of WFS PM_2.5_ > 250 μg/m^3^. A severe WFS episode is defined as any WFS episode that includes at least one week with a sum of WFS PM_2.5_ > 250 μg/m^3^, which could be a single week or a multi-week episode with one or more weeks over this threshold. The 250 μg/m^3^ value was chosen to capture periods with extreme air quality impacts, such as the highly publicized smoke in eastern North America during 2023 wildfire season [40]. **Metric 7** is the total count of WFS episodes and **Metric 8** is the count of severe episodes over the entire exposure window. **Metric 9** is the longest episode (weeks), meaning the WFS episode with the longest duration between the first and last WFS-impacted week in the episode. **Metric 10** is the worst episode (μg/m^3^), meaning the WFS episode with the highest average WFS PM_2.5_ concentration across all WFS-impacted weeks in the episode. Finally, **Metric 11** is the fraction of cumulative WFS PM_2.5_ from severe episodes (%).

Recovery: This metric describes recovery, capturing the periods of time with little to no WFS exposure. **Metric 12** is the average recovery period (weeks) between WFS episodes, calculated as average length of time between episodes, including periods before the first and after the last episodes in the exposure window.

Note that of the twelve MultiWiSE metrics, ten are calculated using only the estimates of weekly WFS PM_2.5_, while two metrics (2 and 6) also require data on total PM_2.5_. As such, most of the metrics can easily be calculated for any long time series of daily WFS-specific PM_2.5_ concentrations, such those generated by deterministic, statistical, or blended modeling approaches [29, 41, 42]. A key difference when using pre-existing estimates of WFS PM_2.5_ to calculate the MultiWiSE metrics is that WFS-impacted weeks need to be defined as weeks where the sum of WFS PM_2.5_ > 0 μg/m^3^, rather than defined using the modified Z-scores of total PM_2.5_.

### Hypothetical locations

**Fig. 3:**
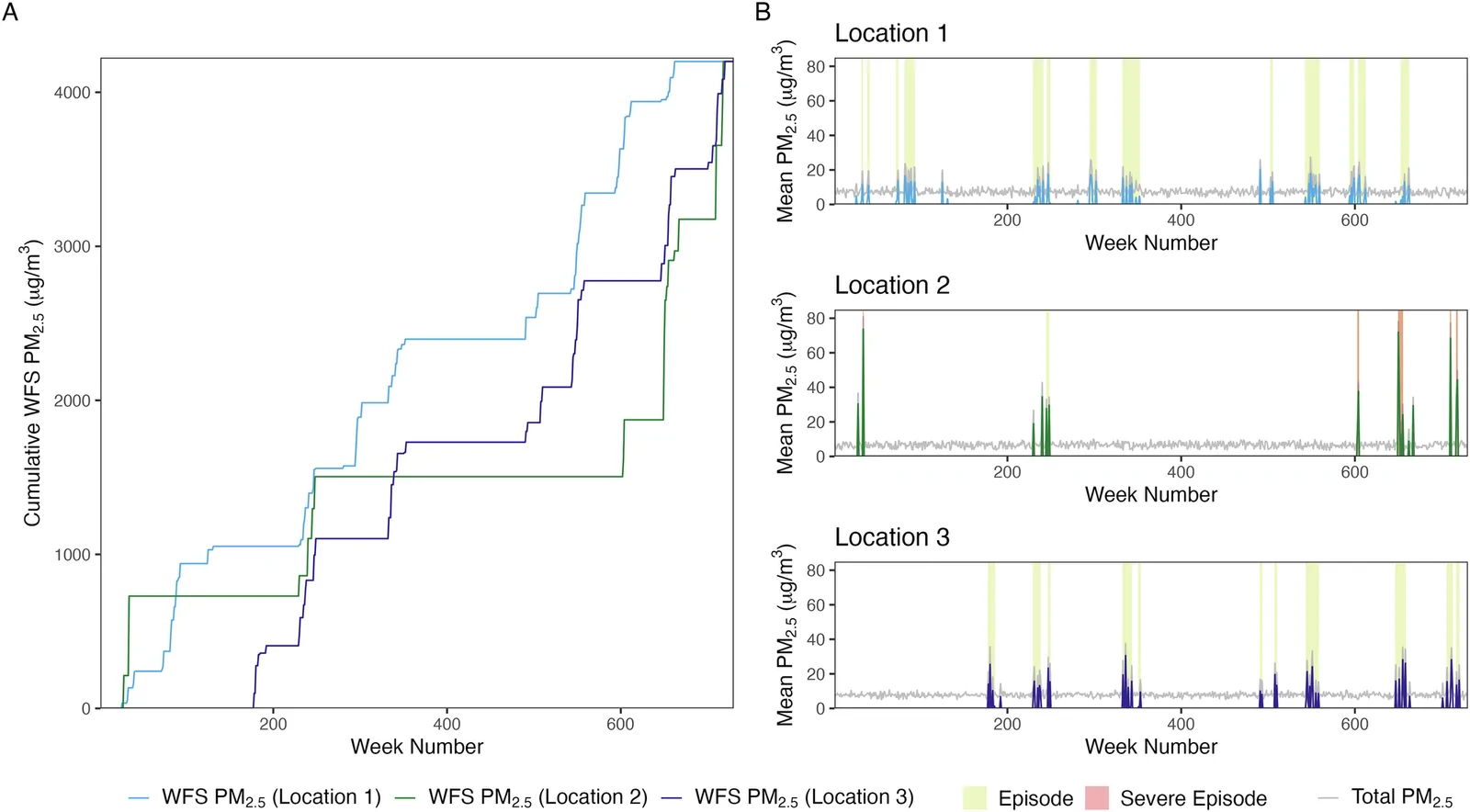
Wildfire smoke (WFS) exposure profiles for three hypothetical locations over a 14-year period. The panels display the **A** cumulative WFS fine particulate matter (PM_2.5_) and **B** weekly average total and WFS PM_2.5_ and WFS episodes for each location. The three locations have the same cumulative WFS PM_2.5_ with different frequencies, intensities, and durations of exposure. The Multiyear Wildfire Smoke Exposure (MultiWiSE) metrics for the three locations can be found in Table 2.

In addition to applying these methods and metrics to data from 2010-2023 across census subdivisions in BC, we applied them to 14 years of weekly PM_2.5_ data generated for the three hypothetical locations described in the introduction (Fig. 3). All three locations had the same cumulative WFS PM_2.5_ over the 14-year period, but the patterns of exposure were different. The first had low levels of WFS PM_2.5_ in most years, the second had high levels in a few years, and the third had moderate levels in approximately half of the years. These hypothetical locations serve to highlight differences between the twelve MultiWiSE metrics in areas with patterns of WFS impacts that are not temporally autocorrelated.

## Results

### Summary of MultiWiSE metrics

Air quality is generally very good across BC, which was reflected in the counterfactual values and non-WFS estimates for 2010–2023. Across the 652 census subdivisions, the mean (range) counterfactual weekly average value was 6.8 (6.1 –7.6) μg/m^3^ (Table 1, Fig. S3), representing the weekly average PM_2.5_ expected in the absence of WFS. The mean weekly average non-WFS PM_2.5_, calculated as the counterfactual on WFS-impacted weeks and total PM_2.5_ on non-WFS-impacted weeks, was 6.9 (6.4–7.7) μg/m^3^ (Table 1, Fig. S4). Across all 730 weeks, non-WFS PM_2.5_ ranged from 3.0 to 24.8 μg/m^3^. In general, there were higher counterfactual and non-WFS PM_2.5_ concentrations in the southern interior (Figs. S3 and S4).

**Table 1:** Summary statistics for the twelve Multiyear Wildfire Smoke Exposure (MultiWiSE) metrics and additional variables used to characterize the frequency, intensity, and duration of episodic exposure to wildfire smoke (WFS) fine particulate matter (PM_2.5_).

Metric	Mean (SD)	Median (IQR)	Range (Min, Max)
*Cumulative Exposure*			
1. Cumulative WFS PM_2.5_ (mg/m³)	5.3 (3.1)	5.3 (5.7)	(0.6, 11.7)
2. WFS Fraction (%)	12.7 (6.6)	13.0 (12.9)	(1.7, 24.5)
*Weekly Exposure*			
3. Average WFS PM_2.5_ (µg/m³)	15.7 (5.9)	15.4 (8.0)	(3.5, 34.9)
4. Any WFS (# weeks)	45.2 (15.2)	48.5 (28.3)	(17, 80)
5. WFS PM_2.5_ > 5 µg/m³ (# weeks)	35.2 (15.8)	37 (31)	(5, 65)
6. Total PM_2.5_ > 25 µg/m³ (# weeks)	11.9 (7.7)	12 (15)	(0, 28)
*Episode Exposure*			
7. WFS Episodes (# episodes)	8.2 (1.9)	8 (2)	(3, 15)
8. Severe Episodes (# episodes)	3.1 (2.0)	3 (4)	(0, 7)
9. Longest Episode (# weeks)	11.8 (4.6)	11.5 (5)	(5, 24)
10. Worst Episode (µg/m³)	33.9 (14.1)	32.6 (14.4)	(4.6, 81.2)
11. WFS from Severe Episodes (%)	56.7 (28.9)	64.2 (42.8)	(0.0, 92.7)
*Recovery*			
12. Average Recovery (# weeks)	78.2 (22.9)	74.2 (21.1)	(41.1, 178.5)
*Additional Variables*			
Average Total PM_2.5_ (µg/m³)	7.9 (0.6)	7.8 (1.0)	(6.6, 9.5)
Average Non-WFS PM_2.5_ (µg/m³)	6.9 (0.2)	6.9 (0.3)	(6.4, 7.7)
Counterfactual Weekly Average (µg/m³)	6.8 (0.2)	6.8 (0.3)	(6.1, 7.6)

Metrics are shown across 652 census subdivisions in British Columbia for 2010-2023.

For total PM_2.5_, the mean (range) weekly average was 7.9 (6.6–9.5) μg/m^3^ (Table 1, Fig. S5) for the 652 census subdivisions, and the range across all 730 weeks was 3.0–217.6 μg/m^3^. These higher values reflect the impacts of WFS as captured by the MultiWiSE metrics (Table 1). The mean cumulative WFS PM_2.5_ for the entire 14-year period (Metric 1) was 5.3 (0.6–11.7) mg/m^3^, and the mean WFS contribution to cumulative total PM_2.5_ (Metric 2) was 12.7 (1.7–24.5) %. The mean WFS PM_2.5_ during WFS-impacted weeks (Metric 3) was 15.7 (3.5–34.9) μg/m^3^, and the number of WFS-impacted weeks (Metric 4) ranged from 17 to 80 of the total 730 weeks between 2010 and 2023. The number of WFS-impacted weeks with average WFS PM_2.5_ > 5 μg/m^3^ (Metric 5) and total PM_2.5_ > 25 μg/m^3^ (Metric 6) ranged from 5 to 65 and 0 to 28, respectively.

For the MultiWiSE metrics characterizing exposure to episodes, the total number of WFS episodes (Metric 7) and severe episodes (Metric 8) ranged from 3–15 and 0–7, respectively, across the 652 census subdivisions over the 14-year period. The longest episode (Metric 9) ranged from 5–24 weeks in length, with a median of 11.5 weeks. The mean (range) weekly average WFS PM_2.5_ during the worst episode (Metric 10) was 33.9 (4.6–81.2) μg/m^3^, and severe episodes accounted for 0.0–92.7% of cumulative WFS PM_2.5_ (Metric 11), with a median of 64.2%. The average recovery period between WFS episodes (Metric 12) ranged from 41.1–178.5 weeks in length (Table 1). The distributions of the MultiWiSE metrics across all census subdivisions can be found in Figure S6.

**Fig. 4:**
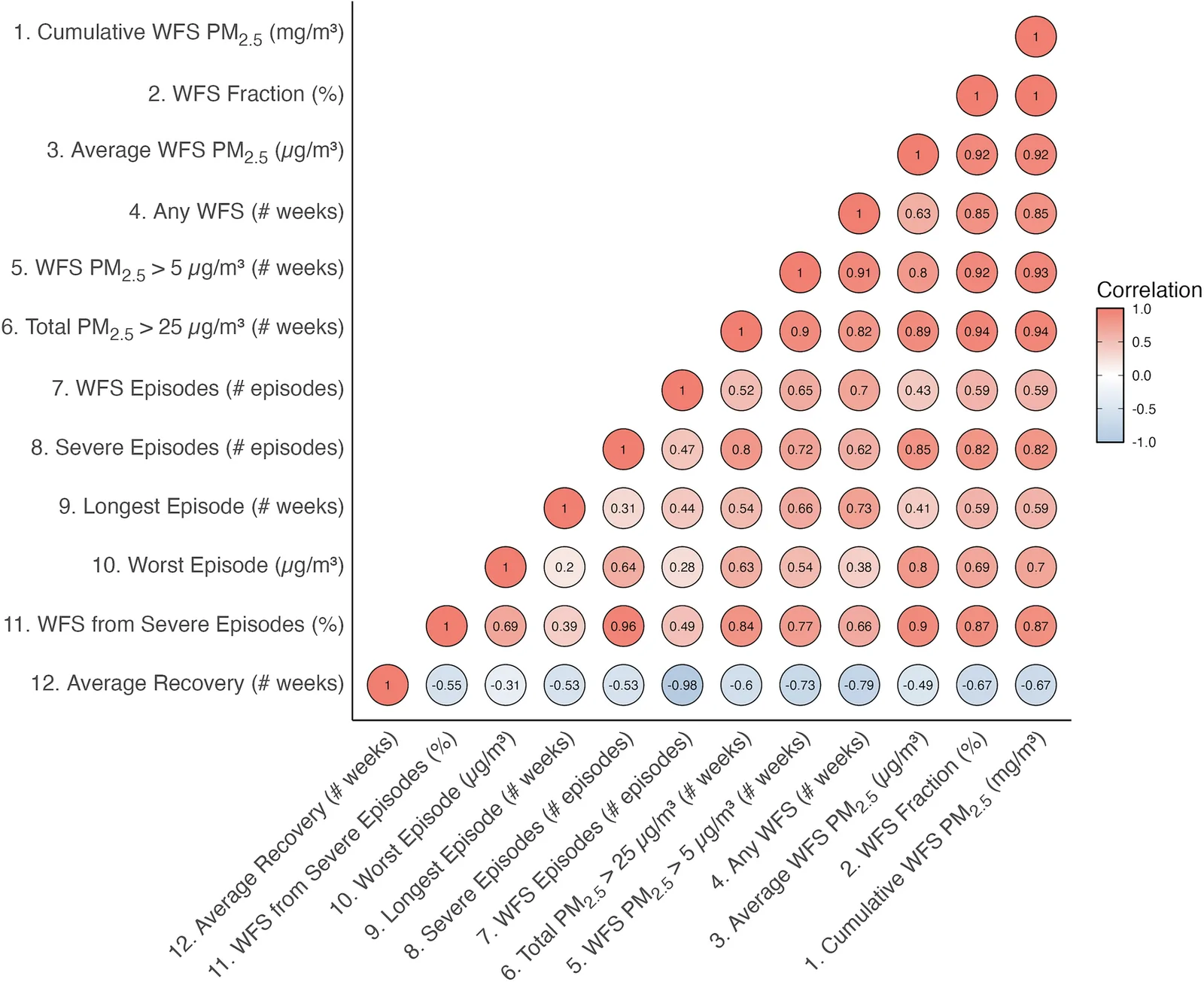
Matrix of Spearman correlation coefficients for the twelve Multiyear Wildfire Smoke Exposure (MultiWiSE) metrics characterizing the frequency, intensity, and duration of episodic exposure to wildfire smoke (WFS) fine particulate matter (PM_2.5_). Data are shown for 652 census subdivisions in British Columbia for 2010–2023.

Overall, the Spearman correlations between the MultiWiSE metrics ranged from –0.98 to 0.99 (Fig. 4). The average recovery period (Metric 12) was the only metric that was negatively correlated with the other metrics, with the values ranging from −0.98 for number of WFS episodes (Metric 7) to −0.31 for the worst episode (Metric 10). All other metrics were positively correlated, with a mean (standard deviation, SD) correlation coefficient of 0.70 (0.20). The weakest correlation (0.20) was between average WFS PM_2.5_ during the worst episode (Metric 10) and the longest episode (Metric 9). The strongest correlation (0.99) was between the two cumulative exposure metrics, cumulative WFS PM_2.5_ (Metric 1) and the fraction of cumulative total PM_2.5_ from WFS (Metric 2), which is to be expected for a region with limited variability in baseline concentrations. In general, correlation was stronger between metrics capturing similar features of multiyear WFS exposure and weaker between those capturing distinct features. For example, the correlation between Metrics 5, 6, and 11, which capture both frequency and intensity of exposure (Fig. 1), ranged from 0.77 to 0.90, while the correlation between these metrics and Metric 7, which captures exposure duration, ranged from 0.49 to 0.65. Correlations between average WFS PM_2.5_ (Metric 3, analogous to the multiyear averages typically used in epidemiologic studies) and the other metrics ranged from 0.41 to 0.92, indicating that the MultiWiSE metrics characterize features of episodic WFS exposure not captured by long-term averages. Across the 66 pairwise comparisons, 23 (35%) had correlation coefficients greater than ±0.80 and 10 (15%) had coefficients greater than ±0.90 (Fig. 4). Conversely, 12 (18%) had correlation coefficients less than ±0.50.

### Temporal patterns in MultiWiSE metrics

When calculated for the first and last 7 years of the 14-year study period, the MultiWiSE metrics show an increase in WFS exposure across the 652 census subdivisions (Table S2). While the mean (range) of non-WFS PM_2.5_ remained largely unchanged (6.8 [6.1–7.7] μg/m^3^ in 2010-2016 compared with 7.0 [6.5–7.9] μg/m^3^ in 2017-2023), the mean WFS PM_2.5_ (Metric 3) increased from 10.1 (3.0–35.5) μg/m^3^ to 17.7 (3.8–40.2) μg/m^3^. Likewise, the mean number of WFS-impacted weeks (Metric 4) and WFS episodes (Metric 7) increased from 10.4 (2–30) and 2.3 (0–6) in 2010–2016 to 34.0 (13–56) and 6.0 (3–9) in 2017-2023. Additionally, the fraction of WFS from severe episodes (Metric 11) increased (17.2 [0.0–83.4] % vs. 62.5 [0.0–100] %) and the average recovery time (Metric 12) decreased (128.6 [47.7–365.0] weeks vs. 48.4 [29.6–87.3] weeks). Similar patterns of increased WFS exposure were observed across all metrics.

### Spatial patterns in MultiWiSE metrics

**Fig. 5:**
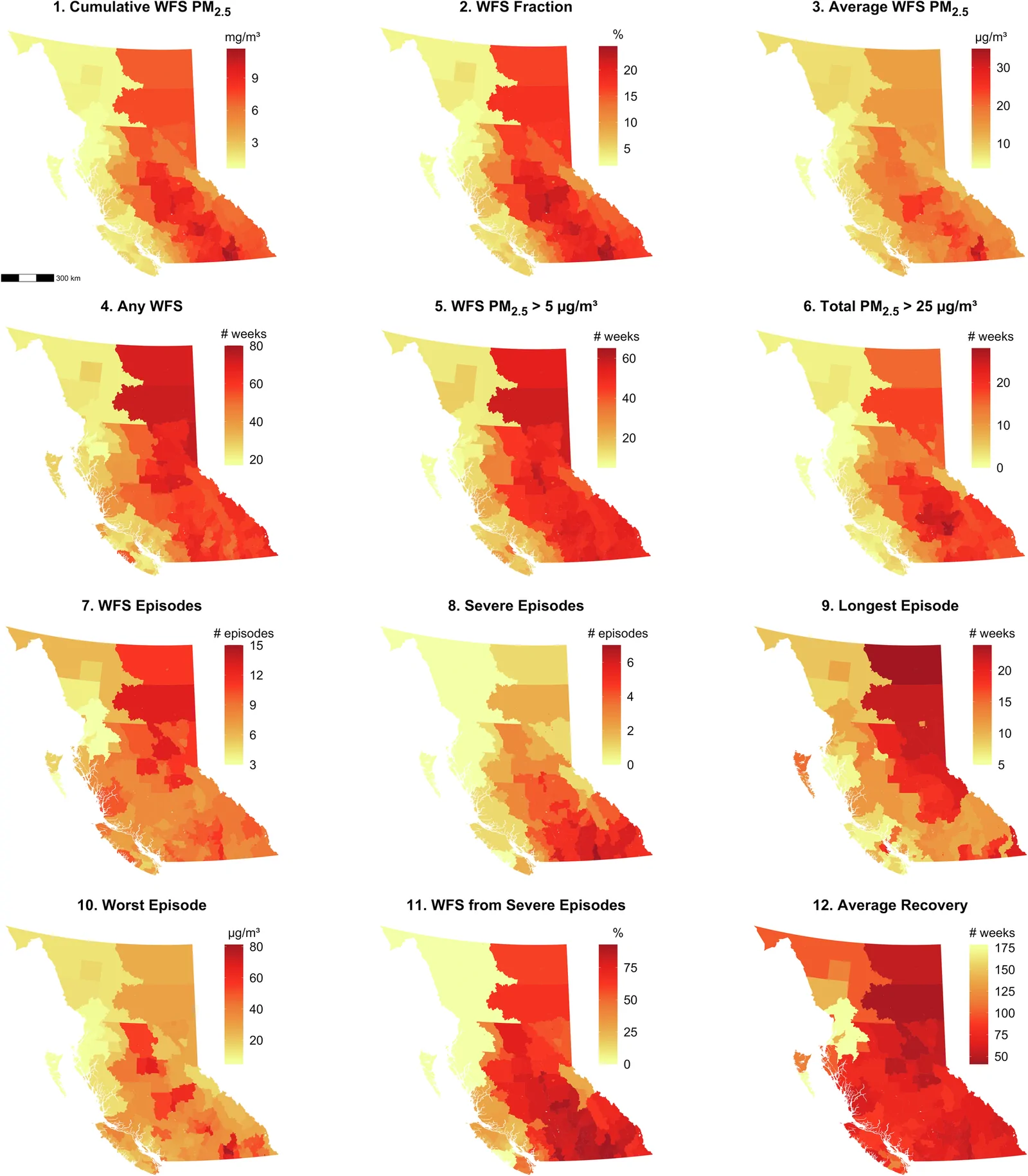
Maps of the twelve Multiyear Wildfire Smoke Exposure (MultiWiSE) metrics characterizing the frequency, intensity, and duration of episodic exposure to wildfire smoke (WFS) fine particulate matter (PM_2.5_). Data are shown for 652 census subdivisions in British Columbia for 2010-2023.

Maps of the twelve MultiWiSE metrics show different patterns of WFS impact across BC (Fig. 5). There is a clear west-to-east gradient for the cumulative and weekly exposure metrics. The highest cumulative WFS PM_2.5_ and WFS fractions (Metrics 1 and 2, which are highly correlated) are seen through the dry interior plateau. The weekly average WFS PM_2.5_ (Metric 3) is more diffuse across the province, with the highest values in the central interior and along the southern interior at the southern border, where WFS from US fires can be transported to BC through the shared intermontane region.

The MultiWiSE metrics focused on WFS episodes generally show different patterns than the cumulative and weekly exposure metrics. In general, there is a north-to-south gradient, with more severe episodes (Metric 8) in the south, but longer episodes (Metric 9) in the north, which can be affected by WFS from long-lasting fires in the northern territories [43]. Conversely, the average recovery period (Metric 12) is shortest in the southern interior, longer in the north and on the coast, and longest for the north coast, which is less impacted by WFS than any other region of the province across all metrics. This area is characterized by dense and protected old growth rainforest, mild temperatures, and low population density.

### Hypothetical locations

The MultiWiSE metrics showed long-term variability in WFS exposure across BC, but they also reflected temporal autocorrelation in the WFS PM_2.5_ data, due to the whole province being affected by the same wildfire activity each season. The data for the three hypothetical locations (Fig. 3) are more temporally distinct, which is apparent in the metric summaries (Table 2). As a reminder, the first location had some WFS in most wildfire seasons, the second had a few severe seasons, and the third was in between. Each location had a 14-year cumulative WFS PM_2.5_ of 4.2 mg/m^3^ with similar cumulative WFS fractions, but all other metrics had distinct values. For example, the average WFS PM_2.5_ concentrations (Metric 3) were 8.6, 35.3, and 13.6 µg/m³, respectively, and the concentrations during the worst episode (Metric 10) were 11.7, 73.7, and 19.4 µg/m³, with average recovery periods (Metric 12) of 45.2, 102.7, and 55.2 weeks (Table 2).

**Table 2:** The twelve Multiyear Wildfire Smoke Exposure (MultiWiSE) metrics characterizing episodic exposure to wildfire smoke (WFS) fine particulate matter (PM_2.5_) in three hypothetical locations (Fig. 3) across a 14-year period.

Metric	Location 1	Location 2	Location 3
*Cumulative Exposure*			
1. Cumulative WFS PM_2.5_ (mg/m³)	4.2	4.2	4.2
2. WFS Fraction (%)	10.3	11.1	9.6
*Weekly Exposure*			
3. Average WFS PM_2.5_ (µg/m³)	8.6	35.3	13.6
4. Any WFS (# weeks)	70	17	44
5. WFS PM_2.5_ > 5 µg/m³ (# weeks)	41	17	40
6. Total PM_2.5_ > 25 µg/m³ (# weeks)	3	14	13
*Episode Exposure*			
7. WFS Episodes (# episodes)	13	6	11
8. Severe Episodes (# episodes)	0	5	0
9. Longest Episode (# weeks)	20	6	15
10. Worst Episode (µg/m³)	11.7	73.7	19.4
11. WFS from Severe Episodes (%)	0.0	70.1	0.0
*Recovery*			
12. Average Recovery (# weeks)	45.2	102.7	55.2

## Discussion

We have developed and applied a set of twelve metrics to characterize episodic multiyear exposure to WFS for use in epidemiologic research on the health effects of longer-term exposures. The metrics can also be used more generally to identify the patterns of WFS exposure over space and time, as demonstrated in Fig. 5 and Table S2. The methods used to generate the MultiWiSE metrics can be applied in any location that has a multiyear time series of daily total (i.e., all-source) PM_2.5_ concentrations without the need for data on wildfire activity or atmospheric conditions. While the MultiWiSE approach was designed to use daily total PM_2.5_ as the starting point, the metrics can be easily adapted when estimates of daily WFS-specific PM_2.5_ are already available. Ten of the metrics can be directly applied to estimates of WFS PM_2.5_ without further modification, and two metrics (2 and 6) can be modified as needed. The metrics are designed for straightforward interpretation, so the epidemiologic results can support public health messages and actions.

When applied to the heavily forested and fire-prone province of BC, the MultiWiSE metrics showed spatial variability across the landmass, with correlations ranging from 0.20 to 0.99, and a mean of 0.70, among the eleven positively-correlated metrics. This suggests they generally capture different features of WFS exposure frequency, intensity, and duration using real-world data. Importantly, the correlations between the metric analogous to the multiyear averages typically used in epidemiologic studies (Metric 3) and all other metrics ranged from 0.41 to 0.92, demonstrating that the MultiWiSE metrics are able to capture features of episodic WFS exposure that are not captured when using annual or multiyear averages. When applied to the three hypothetical locations with different temporal exposure patterns, the metrics also showed clear distinctions between them.

There is growing interest in studying the health effects associated with multiyear WFS exposure, but there are no established methods for characterizing the patterns of WFS exposure that make it different from other sources of air pollution, such as traffic and industry. The MultiWiSE metrics build on previous work by O’Dell et al. [29] to identify WFS-impacted days using typical distributions of total PM_2.5_, and work by Casey et al. [30] and Hahn et al. [31] to describe long-term trends in WFS air quality impacts. Here we extend these ideas by eliminating the need for additional data specific to wildfires, and by developing a comprehensive set of metrics that use estimates of WFS PM_2.5_ to describe cumulative, weekly, and episodic WFS exposures, including severe episodes and average recovery periods.

There have been a limited number of studies on the health effects associated with longer-term exposure to WFS PM_2.5_ [19, 22, 25, 44, 45] and the reported associations are generally consistent with the much larger body of evidence on total PM_2.5_ [17]. Even so, most of the studies have used simple annual or multiyear averages to characterize WFS PM_2.5_ exposure, without explicitly considering the underlying temporal patterns that produced those long-term values. We posit that accounting for these temporal patterns using the MultiWiSE metrics may lead to a more nuanced and biologically grounded understanding of the longer-term health risks posed by WFS. By using them in epidemiologic analyses, investigators may be able to elucidate how the health risks of WFS vary depending on the frequency, intensity, and duration of exposure. This information could help to strengthen public health messaging during WFS episodes.

Consider, for one example, a recent paper on the association between long-term wildfire exposure and cancer incidence in Canada [46]. The authors used wildfire activity as the measure of exposure, but hypothesized that the positive associations with lung and brain cancer were due to carcinogenic pollutants such as WFS, rather than the presence of fire. There are different biological mechanisms underlying these cancers, and it is possible that different features of WFS exposure (e.g., total number of WFS-impacted weeks vs. percent of WFS from severe episodes) would be associated with each. The analyses could be repeated using a subset of the MultiWiSE metrics, testing which ones are best fitted to the data with statistical methods such as the Akaike Information Criterion (AIC) [47]. Furthermore, the analyses could easily be adjusted for the non-WFS PM_2.5_ component, or stratified by different recovery periods to evaluate whether longer recovery times are associated with attenuated effects. The MultiWiSE approach could also be used to explore the effects of WFS compared with the effects of background air pollution. A similarly flexible approach is possible with any exposure window and health outcome of interest, and could be used to build on the existing studies that have characterized longer-term WFS exposure using annual or multiyear averages.

While the MultiWiSE metrics can be flexibly used to characterize multiyear WFS exposure in a variety of contexts, we recommend applying them with the following considerations in mind. First, only a subset of the 12 metrics should be used in a given epidemiologic analysis to avoid multiple comparisons. To select the appropriate metrics, we recommend combining a priori hypotheses on which features of WFS exposure (frequency, intensity, and duration) may be most biologically relevant to the health outcome of interest with information on the correlations between the metrics in the study population. By selecting a subset of relevant, non-overlapping metrics, epidemiologic analyses can better elucidate which features of episodic WFS exposure carry the greatest risk. Furthermore, some variables, such as recovery time (Metric 12) can be considered as effect modifiers. Additionally, although conventional multiyear averages do not capture the dynamic nature of WFS, analyses should consider using them (i.e., Metric 3) to characterize WFS exposures, in addition to the more novel metrics, to enable comparison with existing evidence.

Because the identification of WFS-impacted weeks and WFS PM_2.5_ relies heavily on the counterfactual value and modified Z-scores, we recommend using at least one full year of PM_2.5_ data from a smoke-impacted year to reliably estimate these values. In the example provided here, the same exposure window was used to calculate both the counterfactual and modified Z-scores and the MultiWiSE metrics. However, for use cases where the exposure window of interest is shorter than one year (e.g., pregnancy), a different time period can be chosen to calculate the counterfactual and modified Z-scores (e.g., a set number of years prior to birth).

There are four key strengths to the proposed MultiWiSE metrics. First, the MultiWiSE approach requires only a long daily time series of PM_2.5_ measurements or estimates to generate. Many studies on WFS exposure also use information from wildfire databases, satellites, or mechanistic models to support separating WFS from total PM_2.5_ [48]. While there is value in these approaches, they can also lead to greater complexities associated with accessing, preparing, and using more data, including limitations related to missing data and the spatial and temporal coverage. Here we use entirely data-driven methods to separate WFS from total PM_2.5_, and the results suggest the approach was successful in BC and three hypothetical locations. Second, the metrics are designed for simple epidemiologic interpretation, meaning they can support direct knowledge translation for public health [49]. For example, analyses might show that episode duration is more important than the total number of WFS episodes for some outcomes, and this could be used to support practice in areas where long-lasting events are more common. Third, the ability to capture frequency, intensity, and duration provides opportunities to examine the relevance of different features of longer-term WFS exposure to specific health endpoints and populations. For example, exposure intensity may be most relevant during periods of rapid growth and development, exposure frequency may be most relevant during aging, and exposure duration may matter most for biomarkers or sub-clinical endpoints. Finally, because of their simplicity, the MultiWiSE metrics can be relatively easily calculated for any location and by anyone (more details on this below), potentially making WFS epidemiology more accessible to a broader number of investigators. The MultiWiSE metrics support the critical need to build evidence on the health effects of WFS to better understand the public health risk [50] and motivate shifts in public health policy and practice [51].

There are, of course, also limitations to the MultiWiSE metrics. Importantly, some of the metrics were highly correlated when applied to BC, meaning they were not consistently capturing unique features of WFS in this context. For example, the correlation between cumulative WFS PM_2.5_ and WFS fraction of cumulative total PM_2.5_ was 0.99, which is to be expected in a region with low and invariable baseline concentrations (Fig. S4). These two metrics were also highly correlated (0.92–0.94) with the number of WFS-impacted weeks where PM_2.5_ exceeded defined thresholds (Fig. 4), potentially suggesting that not all four metrics will ultimately be needed. This leads to the second limitation, which is that the metrics have not yet been tested in epidemiologic analyses. Until they have been applied in our research and, ideally, adopted by other users, we cannot know which are the most useful for understanding multiyear WFS exposure and the associated health effects. Finally, the parameters used to define the counterfactual values, WFS-impacted weeks, and episodes may lead to some WFS exposure misclassification, especially during weeks with relatively low total PM_2.5_ concentrations. Likewise, these parameters may lead to greater exposure misclassification in regions or time periods with other highly variable sources of PM_2.5_ during wildfire season, or with little to no WFS impacts. All these parameters can be modified as appropriate, and such misclassification is unlikely to have significant impacts on multiyear epidemiologic studies in WFS-impacted regions.

The MultiWiSE metrics provide a straightforward, data-driven approach for characterizing the health-relevant features (frequency, intensity, and duration) of multiyear, episodic WFS exposure and can be calculated using any long time series of daily PM_2.5_ data. At this early stage of evidence development, we hope the MultiWiSE metrics provide a useful framework for epidemiologic studies to further elucidate the health effects of longer-term WFS exposure. Our research team plans to use the metrics in a series of studies on multiyear WFS exposure and respiratory outcomes across Canada, including lung function decline, and incident asthma, chronic obstructive pulmonary disease, and lung cancer. This will provide further insight into the most useful metrics for different endpoints with different aetiologies, and the results will be reported in future publications. We hope other teams will also use the metrics, which we will support by developing online tools to easily calculate all metrics from a given time series of daily average total PM_2.5_ values and by sharing the code to enable investigators to adapt the MultiWiSE metrics to their specific datasets and research needs. Exposure to WFS PM_2.5_ is fundamentally different from exposure to PM_2.5_ from other sources, and the MultiWiSE metrics may provide more nuanced, biologically relevant, and actionable research into the health effects of prolonged and repeated exposure in the changing climate.

## Supplementary information

## Data Availability

The code and data used to generate the MultiWiSE metrics for British Columbia census subdivisions can be found at: https://github.com/sfu-fhs-cleland/MultiWiSE_Metrics_Manuscript. A dashboard that allows for the calculation of the MultiWiSE metrics using any multiyear time series of daily total (i.e., all-source) PM_2.5_ can be found at: https://sfu-fhs-cleland.shinyapps.io/MultiWiSE-Dashboard/.
