# Supplementary material for: Multiyear wildfire smoke exposure (MultiWiSE) metrics: a data-driven approach to characterizing episodic PM2.5 exposures for epidemiologic research
Source: J Expo Sci Environ Epidemiol. 2026 Apr 13;36(5):860–70. doi: 10.1038/s41370-026-00876-5 (PMC13538030; doi:10.1038/s41370-026-00876-5)
Supplement: Supplementary file 1 [file 41370_2026_876_MOESM1_ESM.pdf]

**Supplementary Information**

# Multiyear Wildfire Smoke Exposure (MultiWiSE) metrics: A data-driven approach to characterizing episodic $PM_{2.5}$ exposures for epidemiologic research

*Stephanie E. Cleland[1,2,3*], Olivia Qiong[3,4], Michael Brauer[4], Sarah B. Henderson[3,4]*

[1] Faculty of Health Sciences, Simon Fraser University, Burnaby, BC

[2] Legacy for Airway Health, Vancouver Coastal Health Research Institute, Vancouver, BC

[3] Environmental Health Services, BC Centre for Disease Control, Vancouver, BC

[4] School of Population and Public Health, University of British Columbia, Vancouver, BC

* Corresponding author: stephanie_cleland@sfu.ca

**Table S1.** The twelve Multiyear Wildfire Smoke Exposure (MultiWiSE) metrics and additional variables characterizing episodic exposure to wildfire smoke (WFS) fine particulate matter ($PM_{2.5}$) in the Kitimat-Stikine C and Vernon census subdivisions between 2010-2023. The $PM_{2.5}$ exposure profiles and locations of these census subdivisions can be found in Figures 2 and S2, respectively.

| **Metric** | **Kitimat-Stikine C** | **Vernon** |
|---|---|---|
| *Cumulative Exposure* | | |
| 1. Cumulative WFS $PM_{2.5}$ (mg/m³) | 0.8 | 8.8 |
| 2. WFS Fraction (%) | 2.2 | 19.8 |
| *Weekly Exposure* | | |
| 3. Average WFS $PM_{2.5}$ (µg/m³) | 6.1 | 24.0 |
| 4. Any WFS (# weeks) | 18 | 53 |
| 5. WFS $PM_{2.5}$ > 5 µg/m³ (# weeks) | 9 | 49 |
| 6. Total $PM_{2.5}$ > 25 µg/m³ (# weeks) | 0 | 19 |
| *Episode Exposure* | | |
| 7. WFS Episodes (# episodes) | 3 | 11 |
| 8. Severe Episodes (# episodes) | 0 | 6 |
| 9. Longest Episode (# weeks) | 8 | 11 |
| 10. Worst Episode (µg/m³) | 7.3 | 40.7 |
| 11. WFS from Severe Episodes (%) | 0.0 | 88.5 |
| *Recovery* | | |
| 12. Average Recovery (# weeks) | 178.5 | 55.8 |
| *Additional Variables* | | |
| Average Total $PM_{2.5}$ (µg/m³) | 6.9 | 8.7 |
| Average Non-WFS $PM_{2.5}$ (µg/m³) | 6.8 | 7.0 |
| Counterfactual Weekly Average (µg/m³) | 6.8 | 6.9 |

**Table S2.** Summary statistics for the twelve Multiyear Wildfire Smoke Exposure (MultiWiSE) metrics and additional variables for first 7 years (2010-2016) and last 7 years (2017-2023) of the study period across 652 census subdivisions in British Columbia.

| | **2010 - 2016** | | | **2017 - 2023** | | |
|---|---|---|---|---|---|---|
| **Metric** | Mean (SD) | Median (IQR) | Range (Min, Max) | Mean (SD) | Median (IQR) | Range (Min, Max) |
| *Cumulative Exposure* | | | | | | |
| 1. Cumulative WFS $PM_{2.5}$ (mg/m³) | 0.7 (0.5) | 0.6 (0.6) | (0.1, 2.3) | 4.6 (2.7) | 4.8 (5.2) | (0.5, 10.3) |
| 2. WFS Fraction (%) | 4.0 (2.5) | 3.3 (3.4) | (0.4, 11.9) | 19.3 (9.6) | 21.2 (18.8) | (2.5, 35.2) |
| *Weekly Exposure* | | | | | | |
| 3. Average WFS $PM_{2.5}$ (μg/m³) | 10.1 (4.4) | 9.3 (5.3) | (3.0, 35.5) | 17.7 (6.7) | 17.3 (8.3) | (3.8, 40.2) |
| 4. Any WFS (# weeks) | 10.4 (5.1) | 10 (6) | (2, 30) | 34.0 (12.0) | 37 (23) | (13, 56) |
| 5. WFS $PM_{2.5}$ > 5 μg/m³ (# weeks) | 6.7 (4.2) | 6 (6) | (0, 28) | 28.1 (12.7) | 29 (25) | (4, 48) |
| 6. Total $PM_{2.5}$ > 25 μg/m³ (# weeks) | 1.2 (1.3) | 1 (2) | (0, 8) | 10.8 (6.9) | 11 (13) | (0, 24) |
| *Episode Exposure* | | | | | | |
| 7. WFS Episodes (# episodes) | 2.3 (1.3) | 2 (2) | (0, 6) | 6.0 (1.1) | 6 (2) | (3, 9) |
| 8. Severe Episodes (# episodes) | 0.3 (0.5) | 0 (1) | (0, 1) | 2.8 (1.8) | 3 (4) | (0, 6) |
| 9. Longest Episode (# weeks) | 5.4 (3.6) | 5 (4) | (0, 22) | 11.0 (4.4) | 11 (6) | (5, 24) |
| 10. Worst Episode (μg/m³) | 15.7 (13.1) | 11.2 (13.6) | (0.0, 68.4) | 34.0 (14.2) | 33.1 (13.5) | (4.6, 84.7) |
| 11. WFS from Severe Episodes (%) | 17.2 (27.7) | 0.0 (41.0) | (0.0, 83.4) | 62.5 (30.1) | 69.0 (43.1) | (0.0, 100.0) |
| *Recovery* | | | | | | |
| 12. Average Recovery (# weeks) | 128.6 (69.1) | 117.7 (93.3) | (47.7, 365.0) | 48.4 (10.6) | 45.4 (12.7) | (29.6, 87.3) |
| *Additional Variables* | | | | | | |
| Average Total $PM_{2.5}$ (μg/m³) | 7.1 (0.3) | 7.0 (0.4) | (6.4, 8.1) | 8.7 (1.1) | 8.8 (1.9) | (6.7, 11.7) |
| Average Non-WFS $PM_{2.5}$ (μg/m³) | 6.8 (0.3) | 6.8 (0.3) | (6.1, 7.7) | 7.0 (0.2) | 6.9 (0.3) | (6.5, 7.9) |
| Counterfactual Weekly Average (μg/m³) | 6.7 (0.3) | 6.7 (0.4) | (5.6, 7.4) | 6.9 (0.2) | 6.9 (0.3) | (6.3, 7.8) |

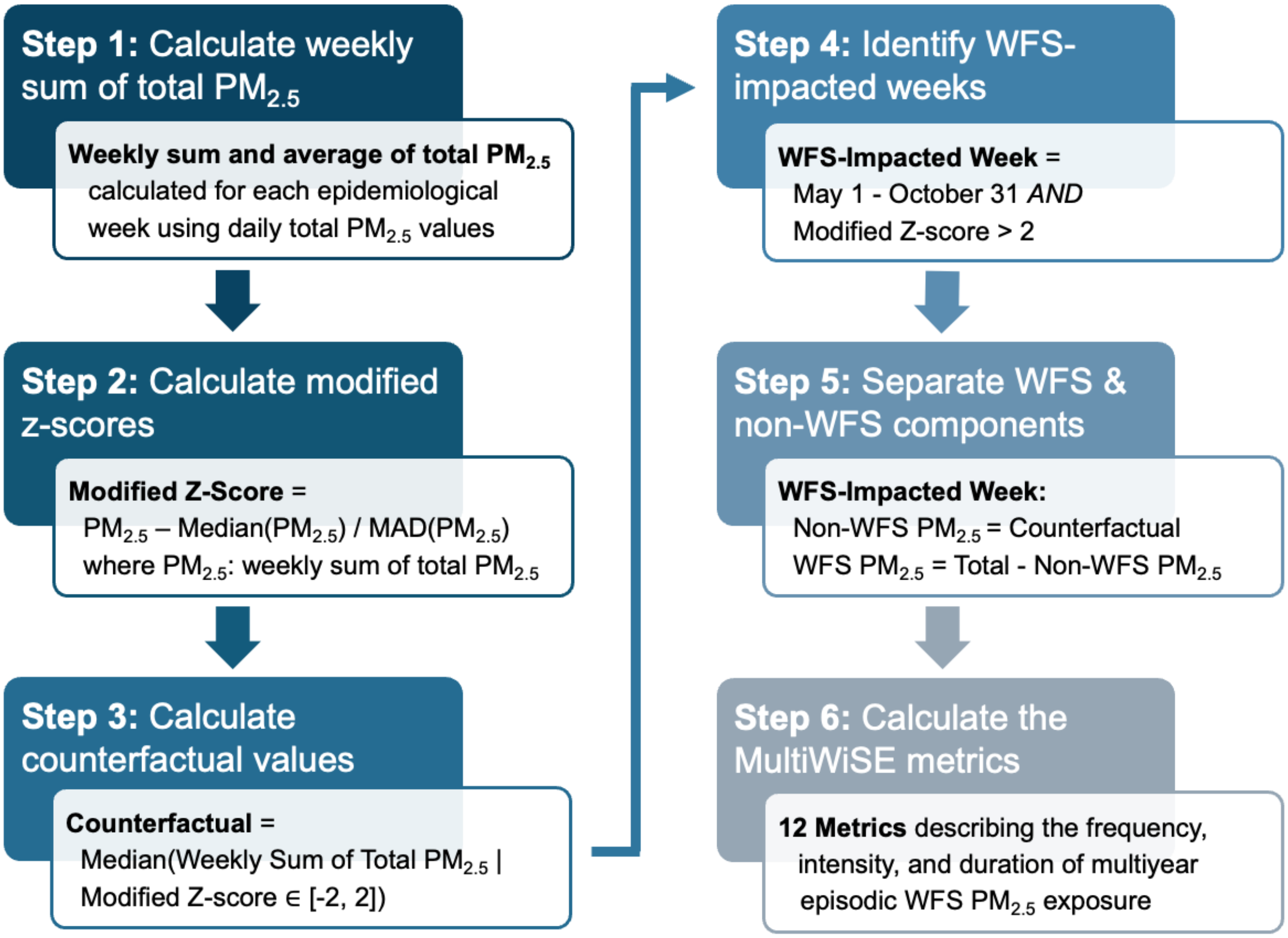


**Figure S1.** The step-by-step methodological approach used to calculate the weekly values, define the counterfactual value, separate total fine particulate matter ($PM_{2.5}$) into non-wildfire smoke (WFS) $PM_{2.5}$ and WFS $PM_{2.5}$ components, and calculate the twelve Multiyear Wildfire Smoke Exposure (MultiWiSE) metrics that characterize the frequency, intensity, and duration of episodic WFS $PM_{2.5}$ exposure.

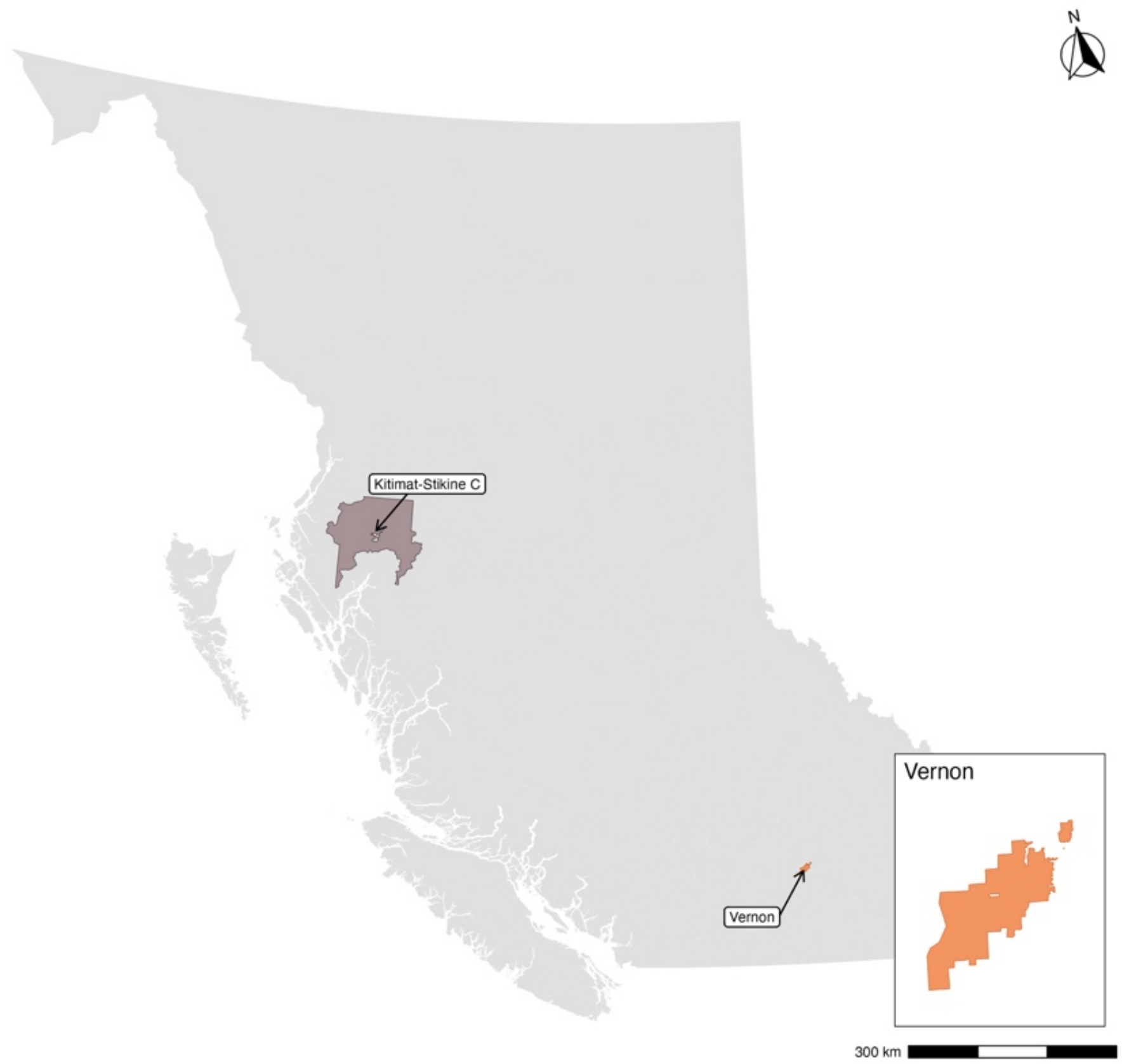


**Figure S2.** The location of the two census subdivisions used as examples in Figure 2 and Table S1.

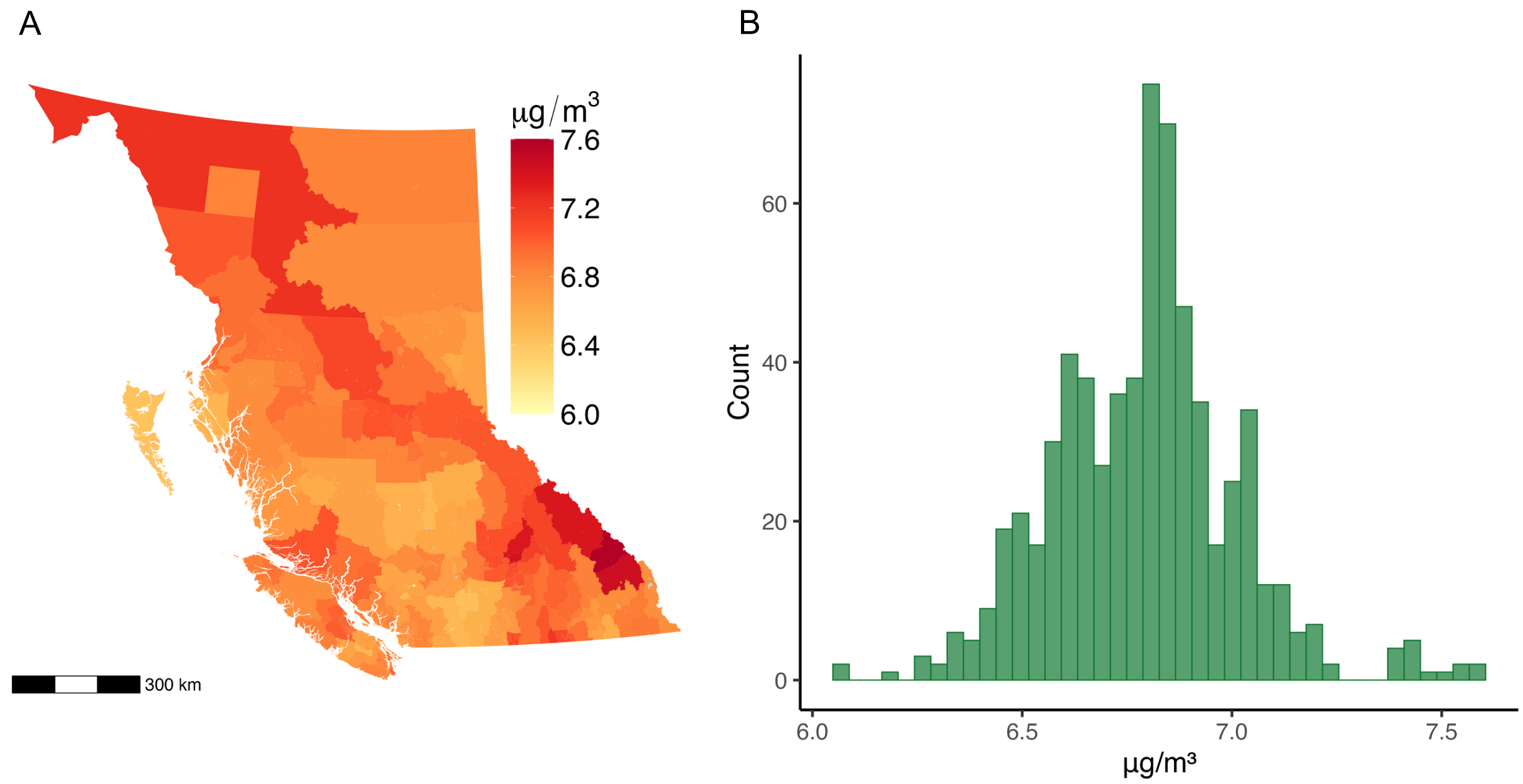


**Figure S3.** (A) Map and (B) histogram of the counterfactual weekly average value, which represents the weekly average fine particulate matter ($PM_{2.5}$) expected in the absence of wildfire smoke. The counterfactual value is calculated as the median of the weekly sum of total $PM_{2.5}$ across all weeks with modified Z-scores between -2 and +2, and the counterfactual weekly average is this value divided by 7. Data are shown for 652 census subdivisions in British Columbia for 2010-2023.

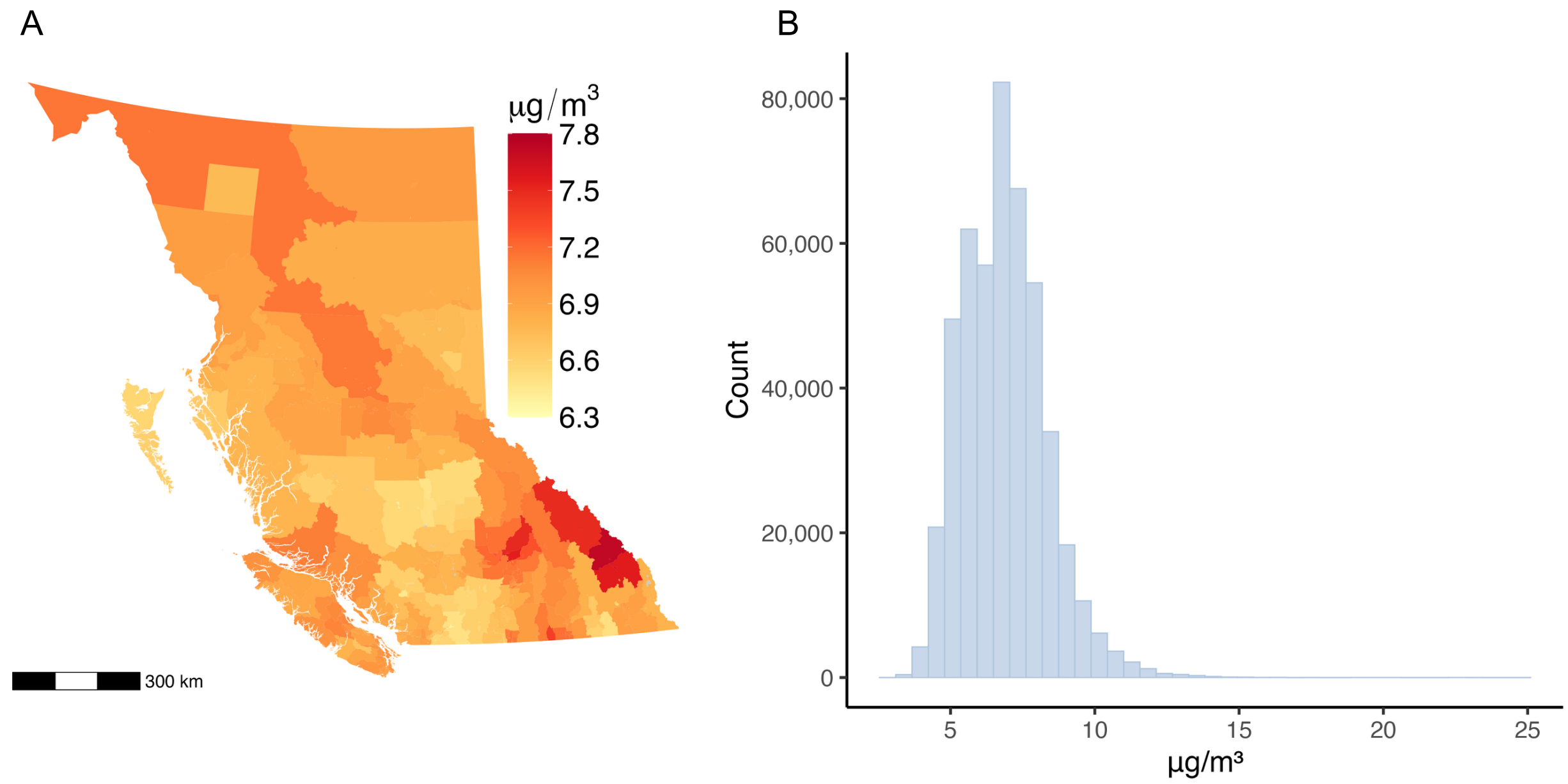


**Figure S4.** (A) Map and (B) histogram of the weekly average non-wildfire smoke fine particulate matter ($PM_{2.5}$). Data shown are (A) the mean of weekly average non-wildfire smoke $PM_{2.5}$ and (B) the weekly average non-wildfire smoke $PM_{2.5}$ across all 730 weeks between 2010-2023 for each of the 652 census subdivisions in British Columbia. Non-wildfire smoke $PM_{2.5}$ is calculated as the counterfactual value on wildfire smoke-impacted weeks and total $PM_{2.5}$ on non-wildfire smoke-impacted weeks.

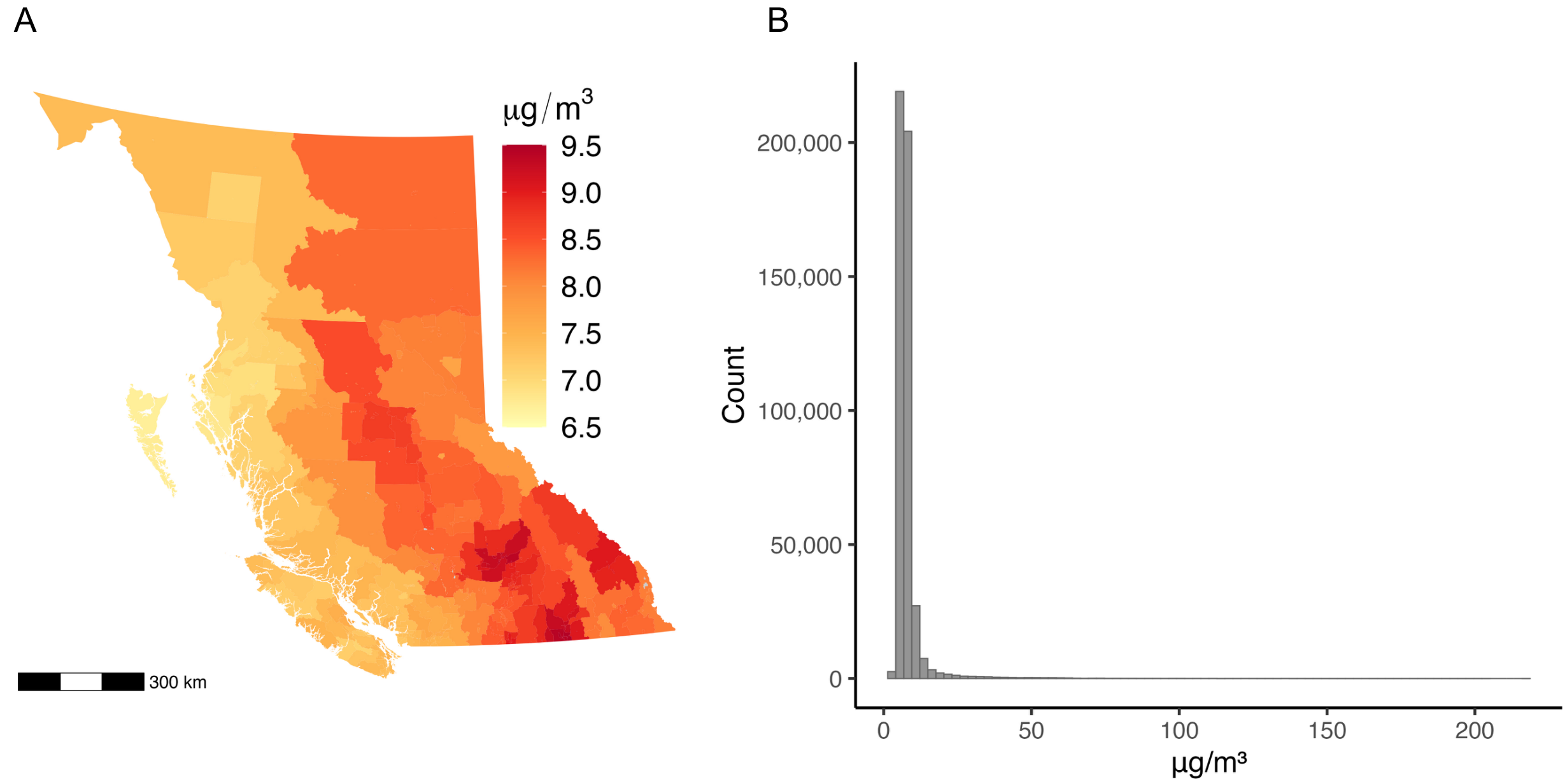


**Figure S5. (**A) Map and (B) histogram of the weekly average total (i.e., all source) fine particulate matter ($PM_{2.5}$). Data shown are (A) the mean of weekly average total $PM_{2.5}$ and (B) the weekly average total $PM_{2.5}$ across all 730 weeks between 2010-2023 for each of the 652 census subdivisions in British Columbia.

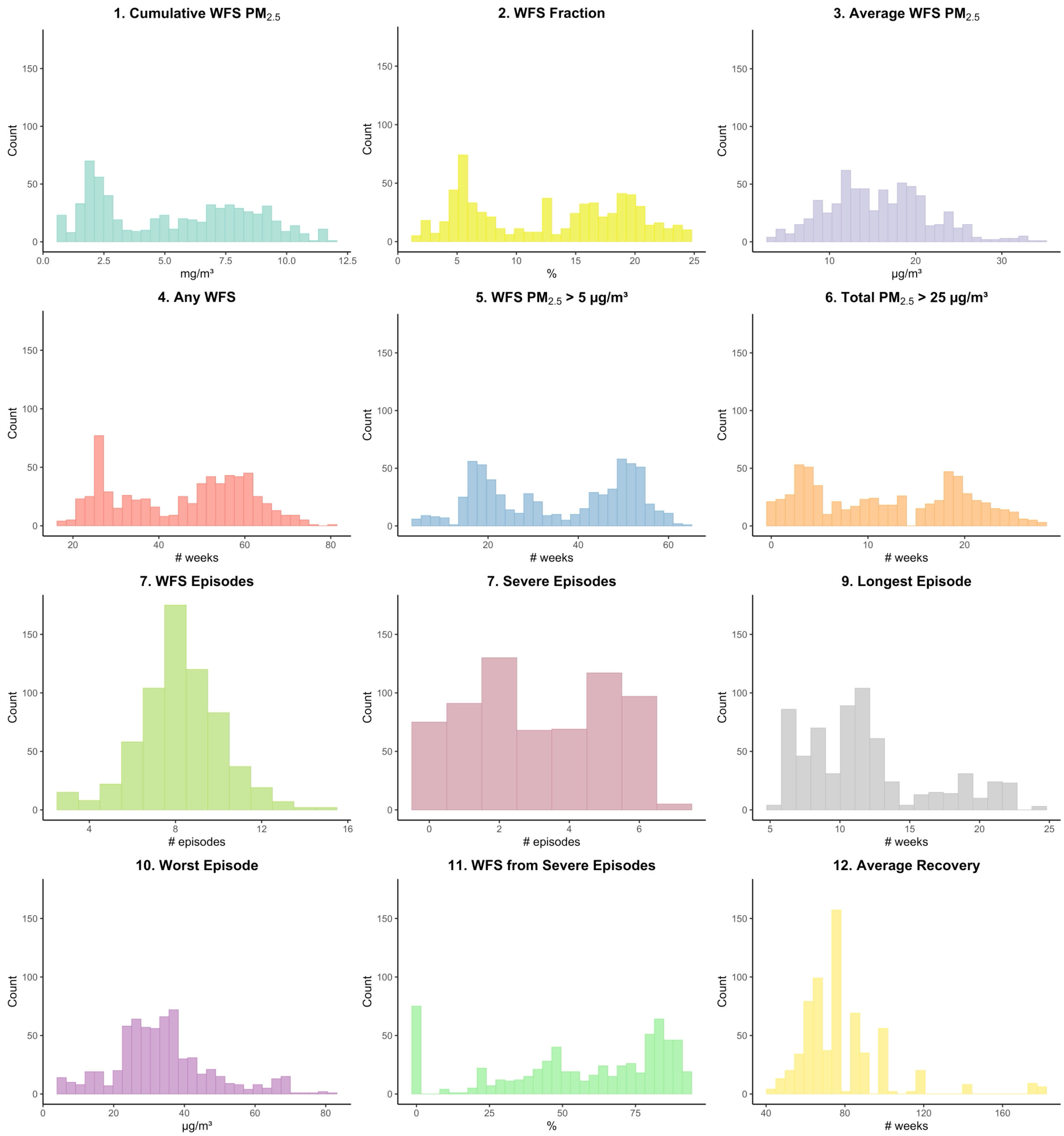


**Figure S6.** Histograms showing the distributions of the twelve Multiyear Wildfire Smoke Exposure (MultiWiSE) metrics across 652 census subdivisions in British Columbia for 2010-2023.
